# Intertwined paths: exploring the link between oral diseases and eating disorders. A comprehensive narrative review

**DOI:** 10.1017/S000711452510500X

**Published:** 2025-09-28

**Authors:** Alessandro Chiesa, Rachele De Giuseppe, Giulia Motta, Francesca Sottotetti, Andrea Scribante, Hellas Cena, Andrea Butera

**Affiliations:** 1 Unit of Dental Hygiene, Section of Dentistry, Department of Clinical, Surgical, Diagnostic and Pediatric Sciences, University of Pavia, Pavia 27100, Italy; 2 Laboratory of Dietetics and Clinical Nutrition, Department of Public Health, Experimental and Forensic Medicine, University of Pavia, Pavia 27100, Italy; 3 Unit of Orthodontics and Pediatric Dentistry, Section of Dentistry, Department of Clinical, Surgical, Diagnostic and Pediatric Sciences, University of Pavia, Pavia 27100, Italy; 4 Clinical Nutrition and Dietetics Unit, ICS Maugeri IRCCS, Pavia 27100, Italy

**Keywords:** Oral health, Oral hygiene, Eating disorders, Anorexia nervosa, Bulimia nervosa, Binge eating disorder, Dental caries, Dental erosion, Periodontal disease

## Abstract

Eating disorders (ED) are psychiatric conditions with profound impacts on physical health, emotional well-being and quality of life. They are associated with reduced employment participation and increased healthcare costs, representing a significant public health concern. Major ED, including anorexia nervosa, bulimia nervosa, binge-eating disorder and other specified feeding and eating disorders, are closely linked to oral health complications, which serve as both diagnostic markers and therapeutic targets in ED management. This narrative review explores twenty-two studies, organised around transdiagnostic behavioural and physiological risk factors, including caloric restriction, purging behaviours, binge episodes and oral hygiene neglect. Evidence indicates that malnutrition, vomiting-induced acid exposure, high intake of cariogenic foods and inconsistent hygiene practices contribute to the deterioration of dental and periodontal health. The review highlights the diagnostic and therapeutic potential of oral assessments in ED management, underscoring the importance of early detection. A dedicated section addresses the role of dental professionals, proposing individualised care pathways and the use of clinical indices such as the Basic Erosive Wear Examination and Schiff air index, alongside emerging tools like tele dentistry. The findings advocate for a multidisciplinary approach, incorporating nutritional support, psychological therapy and targeted dental treatment, which is crucial for developing comprehensive care plans. Such collaboration enhances the effectiveness of interventions, addressing both the physiological and psychological dimensions of ED to improve patient outcomes.

Eating disorders (ED) are complex psychiatric conditions that severely impact individuals’ physical health, emotional well-being and quality of life^(1)^. According to the American Psychiatric Association^(1)^, ED encompass a range of behaviours centred around dysfunctional eating habits and attitudes toward body weight and shape. The main categories, as outlined in the Diagnostic and Statistical Manual of Mental Disorders (DSM-5), include anorexia nervosa (AN), bulimia nervosa (BN), binge-eating disorder (BED) and other specified feeding and eating disorders (OSFED)^(2)^. These conditions lead to physical, mental and social impairments and are associated with the highest rates of cause-specific mortality among mental health conditions^(3,4)^; compared with individuals without ED, those affected experience reduced employment participation, increased absenteeism and presenteeism, higher healthcare and informal care costs and lost lifetime earnings for those who die prematurely^(5)^. The diagnosis of ED involves a comprehensive assessment that includes psychological, behavioural and physiological criteria^(6)^. Clinicians use the DSM-5 criteria, which emphasise patterns of disordered eating, intense fear of weight gain and undue influence of body image on self-worth^(1)^. Psychological assessments often reveal comorbidities, such as anxiety or depression, which further complicate treatment^(7)^. Diagnostic tools may also include physical examinations, laboratory tests and nutritional assessments to evaluate the extent of malnutrition and its physiological impact^(8)^.

Currently, ED represent a significant public health issue, with increasing prevalence globally^(9)^. It has been recently reported that among 12 mental health disorders, ED have experienced the second largest absolute increase in incidence, with age-standardised rates rising from 175·83 per 100 000 in 1990 to 216·02 per 100 000 in 2019, surpassed only by depressive disorders^(10)^.

The global burden of ED increased across all countries, independent of levels of the socio-demographic index; from 1990 to 2019, the disability-adjusted life-years increased for ED across all world regions, particularly in South, East and Southeast Asia^(10)^. The burden on public health is also exacerbated by the associated healthcare costs, long-term treatment requirements and the impact on productivity and social welfare^(11)^. In addition, there is a noted stigma surrounding ED, which often hinders early diagnosis and intervention, further complicating public health efforts aimed at prevention and treatment^(12)^.

Among the ED contributing to the rising incidence, AN stands out due to its severity, chronic course, and substantial impact on individuals and healthcare systems^(13)^. AN affects an estimated 1–4 % of women, with peak onset typically occurring between the ages of 14 and 17^(14)^. In 2019, the years of life lost attributable to AN were calculated at 1674·95^(10)^, reflecting its substantial impact on public health. AN is frequently comorbid with depressive disorders, anxiety disorders, as well as obsessive-compulsive disorder^(15)^, adding to the overall disease burden and placing a significant emotional toll on families and caregivers. The disorder often follows a chronic course, leading to long-term disability; notably, it has been recently reported^(16)^ that only about 30 % of individuals with AN achieved recovery after nine years.

BN, another well-documented ED, is characterised by recurrent episodes of binge eating followed by compensatory behaviours such as self-induced vomiting (SIV) or laxative misuse^(1)^. Individuals with BN usually maintain a normal body weight but exhibit pronounced self-criticism and distorted self-evaluation based on body image^(17)^. These psychological features are closely tied to self-esteem and social functioning. Regionally and nationally, the burden of BN has been found to correlate positively with socio-economic development levels^(18)^. Although females generally bear a higher burden of BN, recent trends show that males are experiencing faster growth rates in diagnosed cases. Projections indicate that, compared with 2021, the age-standardised incidence rate of BN will increase slightly by 2030 in both sexes, with a marginally greater rise in males (0·76 %) than in females (0·24 %)^(18)^.

Until 2019, AN and BN were the only ED recognised as contributors to the global burden of disease^(5)^. This was largely due to the historical focus of epidemiological studies on these two disorders, often overlooking others such as binge BED and OSFED, which were formally added to diagnostic classification systems only in 2013, having previously been included under the DSM-IV category of ED not otherwise specified (EDNOS)^(5)^. BED is characterised by the compulsive consumption of large amounts of food, often accompanied by a sense of loss of control but without compensatory behaviours to prevent weight gain^(1)^. BED is closely linked to conditions such as obesity, hypertension, type 2 diabetes and CVD, which significantly impact both physical health and the overall healthcare burden^(19)^. OSFED encompasses several distinct syndromes and includes atypical or subclinical forms of ED, with symptoms that do not fully meet the diagnostic criteria of other ED^(20)^. Research indicates that BED and OSFED are among the most prevalent ED, with up to half of individuals in treatment for an ED diagnosed with either BED or OSFED^(5)^.

The multifaceted nature of ED necessitates a multidisciplinary approach involving healthcare professionals from psychiatry, psychology, nutrition and other relevant fields such as oral health practitioners^(21,22)^.

The WHO defines oral health as the condition of the mouth, teeth and orofacial structures that allow individuals to perform essential functions such as eating, breathing and speaking, and includes psychosocial dimensions such as self-confidence, well-being and the ability to socialise and work without pain, discomfort and embarrassment^(23)^. Integrating dental and oral health professionals into the treatment framework is particularly crucial, as ED are often associated with oral pathologies, including dental erosion, xerostomia, periodontal disease and dental caries^(24)^. These pathologies result from malnutrition, acid erosion due to vomiting and neglect of oral hygiene, which can compound the individual’s health complications^(25)^.

As for ED, untreated oral diseases affect half of the world’s population and share determinants and risk factors with other most common non-communicable diseases, globally impacting approximately 3·5 billion people across diverse age groups, increasing, particularly in low- and middle-income countries^(26)^. The WHO Commission on the Social Determinants of Health has emphasised the importance of socio-economic, political and environmental health factors exposed to individuals throughout their lives and recognised the need to prioritise oral health within global health agendas^(5)^.

Early detection and intervention are critical for recovery from ED; thus, dental practitioners play an essential role in the recovery process, as they are often the first health professionals to identify signs and symptoms of disordered eating. Given the vital role oral health care providers can have in the early identification, referral and case management of patients with ED, they must acquire comprehensive knowledge of the oral complications associated with these disorders^(27)^.

Building upon the preceding considerations, this narrative review aims to elucidate the intricate relationship between various ED, including AN, BN, BED and OSFED, and a range of oral health complications such as dental erosion, gingivitis, cavities, burning tongue, difficulty in plaque control, tooth sensitivity, increased vulnerability to bacterial colonisation, xerostomia and altered saliva composition. Specifically, recognising the transdiagnostic nature of ED, wherein individuals may transition across diagnostic categories over time^(28)^, the review seeks to identify the behavioural and physiological mechanisms that underpin oral health complications in populations affected by ED. By adopting a risk-factor-oriented approach, this review intends to clarify how shared and disorder-specific behaviours contribute to the deterioration of oral structures and functions, to inform clinical practice and enhance multidisciplinary prevention and treatment strategies.

## Approach to literature selection and thematic data synthesis

This narrative review was conducted by identifying, selecting and synthesising peer-reviewed literature on the oral health consequences of ED. Given the considerable diagnostic overlap and frequent shifts in clinical presentation observed in individuals with ED, the analysis was structured around transdiagnostic behavioural and physiological risk factors, rather than being organised solely by diagnostic category. Although this is not a systematic review, a structured approach was employed to ensure coherence in both the selection of the literature and the presentation of findings.

The thematic organisation by behavioural and physiological risk factors was designed to enhance interpretability and provide a comprehensive understanding of the multifaceted relationship between ED and oral health. Therefore, searches were conducted in the electronic databases PubMed and Web of Science using the following keywords in various combinations: ‘oral health’, ‘eating disorders’ (including specific types such as ‘anorexia nervosa’, ‘bulimia nervosa’ and ‘binge eating disorder’), ‘dental caries’ and ‘dental erosion’; Boolean operators (AND; OR) were also applied to refine the search strategy.

Studies included in the review met specific inclusion criteria: including individuals with ED diagnoses and without age restriction, published in the English language within the last 24 years (2000–2024). Exclusion criteria encompassed *in vitro* and *in vivo* studies; non-English studies conducted before 2000; systematic reviews and meta-analyses, case series and narrative reviews.

A total of 700 studies were initially identified. After applying filters, 223 studies were excluded, leaving 477 for further evaluation. From these, 425 studies were excluded following a detailed review. Of the remaining fifty-two potentially relevant articles, twenty were excluded due to unmet inclusion criteria. A final assessment of thirty-two articles led to the exclusion of ten more, resulting in twenty-two articles included in the review.

The results presented in the selected studies were then thematically grouped into the following four domains, according to behavioural and physiological risk factors (Table 1)

- Caloric restriction and malnutrition^(29–34,36–40)^
- Purging behaviours: Vomiting, laxatives and diuretics^(29,31,33–35,37,41–48)^
- Binge eating episodes^(29,31,34)^
- Oral hygiene neglect^(36,49,50)^

**Table 1.** List of studies grouped according to pathogenic mechanisms that transcend specific ED diagnoses

Pathogenic mechanisms transcending specific ED diagnoses	Number of studies	References
(i). Caloric restriction and malnutrition	11	^(20,22,24,26,29–35)^
(ii). Purging Behaviours: Vomiting, laxatives and diuretics	13	^(20,21,23,25–27,29,32,33,36–40)^
(iii). Binge eating episodes	3	^(20,26,33)^
(iv). Oral hygiene neglect	3	^(24,28,41)^

This thematic organisation was adopted to reduce redundancy and to highlight shared pathogenic mechanisms that transcend specific ED diagnoses. Relevant findings from studies on AN, BN, BED and OSFED (Table 2) were redistributed in the text within these categories without modifying the original scientific content.

**Table 2.** Studies describing the association between ED and oral disorders

Area of Investigation	Authors	Study type	Aim of the study	Study population	ED [Criteria]	Oral evaluation	Results
AN	Shaughnessy *et al.* 2008^(36)^	Cross-sectional study	To investigate the dental and periodontal health of adolescents and young women with Restrictive AN and the relationship between bone mineral density	23 female patients (mean age 17·6 years, age range 14·4–27·2 years)	Restrictive AN [DSM-IV]	Dental/periodontal health: past medical history and dental history; DMFT; plaque score (OHI-S); periodontal disease (GI) and probing depths; screening for oral cancer, dental erosion and other oral pathology. For bone mineral density: Dental panoramic radiographs and bitewing radiographs, according to standard exposure settings.	Dental findings included very good oral hygiene. Gingival recession was present in 43 % of participants (*n* 10). Dental erosion was not seen (mean decayed, missing or filled teeth –DMFT – was 8·6). Weak positive correlation between BMD by MCW on radiographs was observed.
	Paszynska *et al.* 2022^(38)^	Case-control study	Comparing dental health and gingival inflammation level in female adolescents in patients with severe AN-restrictive subtype	117 patients hospitalised in public Psychiatric Unit (mean age 14·9 ± 1·8 years) with severe AN (BMI < 15 kg/m^2^) *vs*. 103 age-matched female dental patients as control group (BMI 19·8 ± 2·3 kg/m^2^, age 15·0 ± 1·8 years) treated in public dental clinic.	AN [DSM 5]	Caries lesions assessed by using DMFT; Erosive wear as BEWE; Gingival condition as BOP; Plaque deposition as PCR.	Patients with AN had: (i). worse dental health than controls (DMFT: 3·8 ± 4·5 *vs*. 1·9 ± 2·1, *P* = 0·005); (ii). more severe erosive tooth wear (BEWE: 18·9 %*vs*.2·9 %, *P* < 0·001); (iii). greater difficulty in plaque control (PCR: 43·8 %*vs*.13·7 %, *P* < 0·001); (iv). higher rates of gingival inflammation (BOP: 20·0 % *vs*. 3·9 %, *P* < 0·001). In the AN group, a significant association was observed between BOP, BEWE and the disease duration, as well as between PCR, decayed teeth and filled teeth (*P* < 0·05).
	Paszyńska *et al.* 2015^(32)^	Cross-sectional study	To evaluate stimulated and resting salivary flow rate and the activity of the salivary enzymes (e.g. amylase, AST, ALT, collagenase, lysozyme, peroxidase, serine and acidic proteases, trypsin) in persons with ED.	28 females with AN (mean ± sd age: 15 ± 2 years) *vs*. 38 female healthy controls (mean ± sd age: 14 ± 1 years)	AN [ICD-10 and DSM-IV]	Clinical examination at the dental clinic to avoid factors affecting or biasing the rate of flow and the pH of saliva. Saliva was collected under both unstimulated and stimulated conditions, according to specific protocols.	Salivary flow rates, both stimulated and unstimulated, were significantly lower (*P* = 0·0005 and *P* = 0·0001, respectively) in the AN group compared with controls. Collagenase and AST activities in stimulated saliva were markedly higher in individuals with AN (*P* < 0·05).
BN	Dynesen *et al.* 2008^(35)^	Case-control study	To study the association between ED and salivary gland function and its relation to dental erosion.	20 women with BN (mean age ± sd: 23·8 ± 4 years) *vs*. 20 healthy controls (with no present or prior history of ED mean age ± sd: 23·1 ± 2 years)	BN [Not specified]	Evaluation of clinical records for dental erosion, fillings, and restorations. Evaluation of intraoral colour photographs at frontal, right and left lateral as well as occlusal views. Evaluation of the degree of dental erosion and impressions in silicone material before preparing casts in epoxy resin. Collection of UWS and SWS in plastic tubes, according to specific protocols. The subjective feeling of dry mouth assessed by the UKU rating scale	UWS flow was significantly lower (*P* = 0·007) in the BN group. Frequency of self-induced vomiting and binge eating did not influence the UWS flow rate. The duration of ED had a significant inverse effect on the salivary flow rate (*P* = 0·019) in the BN group. Hyposalivation (UWS ≤ 0·1 mL/min) was more frequent in the BN group, and the sensation of dry mouth was significantly more pronounced (*P* = 0·003) No differences between the BN group and the control group concerning the SWS flow rate. The experience of oral dryness was significantly (*P* = 0·003) more pronounced in the BN than in the control group. The dental erosion score was significantly higher in the BN than in the control group (*P* = 0·019). The BN group showed a significant relation between the duration of ED in years and dental erosion score (*P* = 0·007)
	Paszyńska *et al.* 2015^(42)^	Controlled clinical trial designed for three age and gender-matched groups	To investigate whether patients with purging-type bulimia and/or non-bulimic patients treated with the serotonin reuptake inhibitor SI-5-HT (fluoxetine) exhibit dental erosion and alterations in specific buffering components of parotid saliva (bicarbonates, phosphates, urea) compared with healthy controls	25 females with BN (group X, mean ± sd age: 21·2 ± 3·2 years) 25 females with bipolar affective disorder without BN (group Y, mean ± sd age: 27·4 ± 6·3 years), receiving 40mg/day of fluoxetine and primary treatment course with mood stabilisers: valproic acid (dose of 800 to 1500 mg) or carbamazepine (dose of 800 to 1200 mg).*vs*. 44 healthy controls (group Z, mean ± sd age: 25·5 ± 4·6 years)	BN [ICD-10 and DSM-IV]	Parotid saliva collected from the subjects using a Lashley cup at rest (chemical stimulation with a 3 % citric acid solution). Clinical examination of dental erosion using the TWI. Measurements of the salivary levels of inorganic phosphates, bicarbonate and urea, as well as salivary pH.	Group X had significant tooth wear in 24 % of subjects Total enamel and dentin loss was observed only in group X. pH and bicarbonate levels were lowest in group X (*P* ≤ 0·05). Bicarbonate concentration was lowest in group X, with significant differences between group X and group Z (*P* = 0·0321) and group Y (*P* = 0·0185). Phosphate levels decreased in all groups after stimulation, with significant differences between group X and Y (*P* ≤ 0·0396) and group Z (*P* ≤ 0·0098). pH and bicarbonate ions: strong correlation in all groups (*P* ≤ 0·0001).
	Blazer *et al.* 2008^(44)^	Cross-sectional study	To examine the salivary composition, and taste perception in patients with EDs starting from the *priori* hypothesis that changes in either taste perception or salivary composition might be associated with the active disease.	26 consecutive female patients with BN (mean ± sd age: 24 ± 7 years) *vs*. 26 healthy female controls matched for age and BMI.	BN [DSM-IV]	Structured questionnaire to evaluate xerostomia, the need for frequent mouth-rinsing, difficulties in masticating/swallowing or communicating, and taste aberrations/burning sensation in the oral cavity. Saliva collection according to the widely accepted procedure	Xerostomia complaints were significantly more common among BN patients than controls (*P* < 0·003). - 31 % of patients reported moderate to severe xerostomia, and 46 % reported mild symptoms. 47 % of patients experienced taste disturbances or burning sensations in the oral cavity, compared with 19 % of controls (*P* < 0·016). No significant differences between patients and controls in the need for mouth rinsing or in difficulties with chewing, swallowing or communication.
	Aframian *et al.* 2010^(46)^	Cross-sectional and case-control study	To compare the PH of oral mucosa in healthy subjects to individuals with GERD, BN and BMS.	GERD group: *n* 26 (12M/14F; (mean ± sd age: 56·3 ± 12·8 years); BMS group: *n* 29 (22M/7F; mean ± sd age: 55·4 ± 13·4 years); BN group: *n* 22 females (mean ± sd age: 27·7 ± 10·6 years) *vs*. 26 (11M/25F) healthy volunteers (mean ± sd age: 52·1 ± 9·1 years)	BN [DSM-IV]	Measurements of the oral surface pH with a flat, glass electrode pH metre.	The BN and GERD groups exhibited significantly lower pH (6·38 ± 00·45 and 6·51 ± 0·32, respectively, *P* < 0·05) compared with the control group (6·8 ± 0·33).
	Manevski *et al.* 2020^(48)^	Prospective observational clinical study	To determine the presence, localisation and degree of dental erosion and the caries experience in patients with purging BN, compared with healthy controls.	30 patients (aged 18–35 years) diagnosed with purging BN for at least 3 years. *vs*. 30 healthy subjects well-matched for age and gender as controls.	Purging (frequent vomiting - minimum 2–3 times/week in acute phase) BN. [Not specified]	Intraoral inspection and assessment of dental status using BEWE and DMFT indexes	Dental erosion was significantly more prevalent and severe in patients with purging BN compared with controls (*P* < 0·05), particularly on the oral surfaces of the teeth. DMFT index values did not differ significantly between patients with BN and controls (*P* = 0·461).
AN, BN, BED, EDNOS/ OSFED	Johansson *et al.* 2012^(29)^	Case-control study	To evaluate the oral health status and assess the prevalence of self-reported symptoms in patients with EDs undergoing treatment at an outpatient specialist clinic	54 patients (50F /4M; mean age 21·5 years; age range: 10–50 years) *vs*. Healthy control group of 54 subjects, matched for sex and age	AN (28 %, *n* 14); BN (14 %, *n* 8); EDNOS (58 %, *n* 32) [Not specified] Among controls, the symptom index of the EDI-2 was used to identify and exclude patients with a risk of having ED	Lip dryness and cracks were assessed visually, parotid swelling was evaluated by inspection and palpation. The VPI and GBI were recorded on buccal, mesiobuccal, and lingual surfaces. Dental caries was examined by visual-tactile inspection and bitewing radiographs. DMFT and DMFS were recorded from clinical and radiographic data. Dental erosion was graded on an ordinal scale; buccal cervical defects were documented without specifying morphologies. Impressions of both arches were made for first molar cupping assessments and intraoral soft tissues.	Patients with ED showed significantly higher ORs for presenting with dental issues (OR = 4·1), burning tongue (OR14·2), dry or cracked lips (OR = 9·6), dental erosion (OR = 8·5), and reduced gingival bleeding (OR = 1·1) compared with healthy controls. The sensitivity and specificity of using these five indicators to classify patients with ED *vs*. controls were 83 % and 79 %, respectively. Patients with vomiting or binge-eating behaviours reported poorer perceived oral health (OR = 6·0) and exhibited greater dental erosion (OR = 5·5) than those without these behaviours. Dental erosion was more prevalent in patients with ED with longer disease duration.
	Panico *et al.* 2018^(41)^	Prospective case-control study	To describe oral lesions in patients with ED	65 female patients with ED (mean age: 21·5 years, age range 12–32 years). *vs*. 65 healthy female controls (mean age 23·21 years, age range 14–31 years)	AN (3 %, *n* 6); BN (71 %, *n* 46); EDNOS (20 %, *n* 13) [DSM-IV]	Oral lesions were recorded through visual examination	In the study group, 94 % presented a total of 112 oral lesions, compared with 18·5 % (12 individuals) in the control group. The prevalence of oral lesions in the study group was significantly higher than in controls (OR 50·8, 95 % CI 15·8–162·9, *P* < 0·0001). The most common lesions included labial erythema, exfoliative cheilitis, orange-yellow palate, hemorrhagic lesions, lip-cheek biting and non-specific oral atrophies; no major salivary gland swelling was detected. The presence of all oral lesions combined was associated with purging behaviours (OR 6, 95 % CI 1·06–34·12, *P* = 0·0414). Labial erythema was more frequent in individuals with BN than in those with AN or EDNOS (*P* = 0·0098) and was linked to self-induced vomiting (OR 4, 95 % CI 1·08–14·77, *P* = 0·0396) and diuretic/laxative use (OR 7·89, 95 % CI 2·18–28·56, *P* = 0·0012). An orange-yellow discolouration of the palate (in 34 % of the study group) was associated with high intake of carotene supplements.
	Uhlen *et al.* 2014^(31)^	Clinical study	To evaluate the distribution and severity of dental erosions in Norwegian patients experiencing SIV	72 patients with ED; only 66 individuals with SIV (63F/3M; mean age 27·7 years; age range 20–48 years) undergoing psychiatric and/or medical outpatient treatment at clinics for ED during 2005 – 2013, were evaluated	BN (*n* 62) AN (*n* 8) BED (*n* 1) EDNOS (*n* 1) [Not specified]	Erosion registered using VEDE system	Forty-six individuals (69·7 %) exhibited dental erosions (19 had lesions only in enamel; 27 had lesions in both enamel and dentine). In 26·1 % of cases, 10 or more teeth were affected, and 9 % had dentine lesions in at least 10 teeth. Most erosions were located on palatal/lingual (41·6 %), occlusal (36·6 %), and buccal (21·8 %) surfaces. Individuals with longer illness durations had a higher incidence of lesions (48·6 % of all erosions and 71·7 % of dentine lesions). 30·3 % of participants showed no lesions, even with SIV for up to 32 years.
	Otsu *et al.* 2014^(43)^	Observational study	To analyse the association between the severity of erosion and the behaviour of patients with ED, with a focus on daily diet and vomiting behaviour.	71 female patients (mean age 31·1 years; age range 17–47 years) with ED	AN (*n* 35; Restricting type = 11, Binge-Eating/Purging type = 24). BN (*n* 27; purging type = 21, non-purging type = 6). EDNOS (*n* 3 and unclear diagnosis in = 6) [DSM-IV-TR] Based on self-induced vomiting behaviour, patients were further classified into vomiting (*n* 50) and non-vomiting (*n* 21) groups.	The occurrence and severity of dental erosion assessed using diagnostic criteria for dental erosion and scored as E1 (slight) to E4 (severe).	Dental erosion was observed in 43 of 50 subjects, engaged in self-induced vomiting, with the palatal surfaces of the maxillary anterior teeth being most frequently affected (81·3 % of subjects). Significant differences were found between subjects with mild *vs*. severe erosion based on their post-vomiting oral hygiene practices. Subjects in the mild erosion group were significantly more likely to consume large amounts of water before vomiting, whereas those in the severe erosion group more commonly consumed carbonated beverages or sweetened foods.
	Pallier *et al.* 2019^(49)^	Case-control study	To assess dental and periodontal health in patients with AN and BN	70 Females (aged <65 years) with an ED diagnosed for at least 5 years and 70 females well-matched for age, without ED as a control group.	AN and BN [M.I.N.I. questionnaire]	Self-reported behaviours regarding oral health. DMFT, BEWE, PI, BOP.	Patients with ED exhibited: more frequent dental visits and toothbrushing than controls (*P* < 0·01); higher plaque index and bleeding on probing levels than in controls (*P* ≤ 0·03). gingival recession greater than 2 mm than in controls (2·3 ± 4·1 % *vs*. 0·0 ± 0·1 %, *P* < 0·01). BN patients showed BEWE score>2 more frequently than AN (76·5 % *vs*. 41·7 %, *P* < 0·01). Patients with AN showed increased plaque index, bleeding on probing and clinical attachment loss relative to those with BN.
	Chiba *et al.* 2019^(37)^	Quantitative cross-sectional study	To investigate periodontal health, alterations in salivary biochemical parameters, and oral health-related quality of life in patients with ED.	30 female patients with ED (mean age ± sd: 31·13 ± 12·72) *vs*. 30 female healthy controls (mean age ± sd: 28·93 ± 9·77 years)	AN and BN [Not Specified]	Oral clinical examinations to evaluate the periodontal condition by CPI. OHIP-14 to assess OHRQoL. Saliva samples to evaluate levels of total protein, ALT, AST, LDH, TBARS and salivary flow rate.	The CPI in the EDs group was significantly worse (*P* < 0·05 for healthy gingival, bleeding on probing, presence of calculus) compared with the controls. The salivary flow rate was higher in the EDs group than in the controls (*P* = 0·0001). The EDs group had markedly higher salivary concentrations of total protein (*P* = 0·0109), AST (*P* = 0·0108), ALT (*P* = 0·0455), and LDH (*P* = 0·0252) than controls. No differences were found in salivary TBARS levels. The OHIP-14 score was higher (*P* < 0·05) in the EDs group than in controls.
	Esteves *et al.* 2020^(45)^	Cross-sectional and case-control study	To investigate and assess the prevalence of oral *Candida spp* in persons with ED.	30 females with AN or BN (mean age ± s e, 32 ± 1·7 years) *vs*. 15 female healthy controls (without oral mucosal lesions and EDs; mean age ± s e, 30·7 ± 2·6 years)	AN (*n* 7) and BN (*n* 13) [DSM 5]	Collection of oral swabs (e.g. from the dorsum of the tongue, palate, buccal mucosae, and supragingival area). Evaluation of decayed, missing, and filled teeth (DMF) index.	Higher isolation rate of non-albicans species (*C. glabrata, C. parapsilosis and C. dubliniensis*) in EDs group with nutritional deficiencies, compared with the control group. In the ED group: yeasts were isolated from the oral cavity in 53 % of patients, resulting in 75 yeast isolates. Of these, 43 isolates were identified by conventional mycological methods: *C. parapsilosis:* 19 isolates; *C. glabrata:* 16 isolates; *Rhodotorula species:* 6 isolates; *C. famata:* 2 isolates. The remaining 32 isolates were presumptively identified as *C. albicans* or *C. dubliniensis* In the control group, only 26·7 % of subjects harboured *C. albicans*.
	Lourenço *et al.* 2018^(33)^	Case-control study	To investigate the oral health status and orofacial problems in outpatients with ED	33 female outpatients (aged between 18 and 50 years) *vs*. 33 healthy women without previous history or risk of suffering of EDs (aged between 18 and 50 years), as control group	AN and BN [DSM 5] Outpatients were also sub categorised into a vomiting group (VG) and a non-vomiting group (NVG)	Questionnaire to assess potential factors influencing oral health. Visual dental examination of all teeth, including third molars. Tooth decay evaluation through visual and probe inspection, with calculation of DMFT and DMFS indexes. Dentin hypersensitivity assessment based on patient self-reports. Periodontal status evaluated on index teeth through probing. Xerostomia assessment based on patient-reported dry mouth and difficulties with oral functions. NSSF measured using a modified Schirmer’s test (MST).	Outpatients with ED reported significantly (multivariate analysis, *P* < 0·05) higher incidence of dental complications, including tooth decay, dental erosion and self-reported dentin hypersensitivity, as well as periodontal disease, salivary issues (hyposalivation and xerostomia) and oral mucosal complications. Dental erosion, dentin hypersensitivity, hyposalivation, xerostomia and angular cheilitis showed strong correlations with vomiting behaviour.
	Johansson *et al.* 2022^(34)^	Cross-sectional study	To explore behaviours and habits in individuals with ED and SIV in relation to oral health.	65 persons with EDs were invited to participate in the study; 54 of those accepted, and an age- and sex matched control group was selected. 17 females of the participants in the ED group who reported SIV during the previous six months are the sample for the present study (mean age of 23 years, age range 16 to 50 years).	EDNOS (*n* 10) BN (*n* 6), AN (*n* 1). [DSM-IV]	Questionnaire related to compensatory behaviours, such as SIV (onset, frequency, purging, technique for induction and symptoms afterwards), binge eating (time aspects, type of food/drinks, and subsequent vomiting), and oral hygiene habits.	Binge eating before SIV occurred in 14 out of 17 patients. Timing and post-vomiting procedures, like tooth brushing (7/17), varied. Binge eating often involves high-calorie (sugar/fat) or acidic foods and drinks. All patients believed vomiting could damage teeth, but only one disclosed their ED to a dentist. 10 patients rated their oral health as good/fairly good, while 7 rated it as not so good/bad/very bad. Media was the primary source of information on ED-related dental effects.
	Chiba *et al.* 2022^(39)^	Case-control study	To evaluate the prevalence/ severity of malocclusion and its impact on OHRQoL and self-reported satisfaction in individuals with ED	60 female patients with EDs (AN or BN, mean ± sd age: 31·13 ± 12·72 years) *vs*. 30 patients without EDs (mean ± sd age: 28·93 ± 9·77 years) as the control population	AN and BN [Not specified]	Dental occlusion evaluated by the DAI. OHRQoL assessed using the OHIP-14 questionnaire. Self-reported satisfaction with the appearance of teeth, speech ability and chewing assessed by interview.	Severe malocclusion (26·67 % of patients) and very severe malocclusion (46·67 % of patients) in the ED group. No abnormality/only mild malocclusion in the control group. The ED group had a significantly higher proportion (*P* < 0·05) of patients with tooth loss, spacing in the incisor region, maxillary and mandibular misalignment Significantly lower (*P* < 0·05) OHRQoL and less satisfaction with appearance of teeth, speech ability and chewing ability in the ED group. Significant positive correlation (*P* < 0·05) between DAI and OHIP-14 scores in the ED group.
	Perlman *et al.* 2008^(47)^	Comparative observational study	To compare the prevalence of psychological, dental and TMD signs and symptoms between young women with ED and an age-matched control group of healthy women, as well as to assess the impact of frequent vomiting on these signs and symptoms within the EDs group.	79 hospitalised females (mean ± sd age: 23·46 ± 3·54 years. Among them, 43 with vomiting (mean age 23·8 ± 3·5 years), and 36 with no vomiting (mean age 23·0 ± 3·5 years) *vs*. 48 healthy females (mean ± sd age: 24·58 ± 3·01 years), as controls.	BN (*n* 29) AN (*n* 24) EDNOS (*n* 16). Several patients had more than 1 diagnosis and were placed into a mixed diagnosis group (*n* 10). [DSM-IV]	Questionnaire referring to TMD symptoms and oral habits investigating limitation in mouth opening, tooth sensitivity in response to mastication, the prevalence of chewing gum, performance of oral habits and awareness of awake bruxism. Clinical examination of TMD signs: range of AMO; range of PMO; presence of clicks and/or crepitation; joint sensitivity; muscle sensitivity to digital palpation Examination of hard tissues inside the oral cavity.	Women with ED exhibited significantly greater: sensitivity to muscle palpation (*P* < 0·001); levels of depression, somatisation, and anxiety (*P* < 0·001); prevalence of intensive gum chewing (*P* < 0·001); occurrence of dental erosions (*P* < 0·001) and attrition (*P* < 0·001) ED patients who engaged in vomiting showed higher muscle sensitivity to palpation compared with non-vomiting patients (*P* < 0·001) and increased emotional and psychological distress (*P* < 0·001).
EDs without specifications	Dehghan *et al.* 2023^(30)^	Pilot cross-sectional clinical study	To examine oral health conditions, behaviours and saliva characteristics associated with tooth erosion in women with EDs, testing the following hypotheses (i). Adult females with EDs exhibit greater severity of tooth erosion than healthy women (ii). Specific health histories, oral health behaviours, oral conditions and saliva characteristics are predisposing risk factors for tooth erosion.	14 female patients with EDs (mean ± sd age: 33 ± 13 years) *vs*. 10 female healthy controls (mean ± sd age: 33 ± 10 years)	Assessment of the presence of ED (active purging in 64 % of the subjects) [DSM 5]	Dental caries status was recorded as DMFT index Tooth erosion was assessed using Lussi’s scoring system for the BEWE, grading severity on facial, lingual, and occlusal/incisal surfaces on a four-point scale. Saliva was collected for flow rate, PH and analysis of buffering capacity	The EDs group had a significantly lower stimulated saliva flow rate (*P* = 0,02) and higher DMFT index (*P* = 0·006) than controls. No significant difference in BEWE scores compared with the controls (*P* = 0·08). Five of the fourteen ED subjects exhibited extensive tooth erosion, which may have been exacerbated by their tooth-brushing behaviour.
	Mazurek *et al.* 2016^(50)^	Survey study	To examine oral health behaviours and the condition of teeth and gums in women with ED.	30 female patients with ED (age range 14–36 years) *vs*. 30 healthy females (control)	Patients with ED [Not specified]	Questions on oral health-related behaviours covered the frequency of tooth brushing, type of toothbrush used, frequency of dental visits, dental anxiety, self-assessment of teeth and gum health and experiences of dry mouth.	Toothbrushing frequency: 40 % of patients brushed more than twice daily, compared with 17·24 % of controls (*P* = 0·01). Change in oral hygiene after illness onset: 56·67 % of patients reported increased oral hygiene care; 40 % of patients reported no change in their oral hygiene routine. Gum health: Significantly more patients reported gum problems compared with controls (*P* = 0·002). Dry mouth sensation: Reported by 56·57 % of patients *vs*. 20 % of controls, a statistically significant difference (*P* = 0·003).
	Garrido-Martínez *et al.* 2019^(40)^	Comparative transversal epidemiological study	To describe the oral and dental health status of subjects with ED, assessing several oral manifestations (e.g. dental alterations, periodontal disorders, soft tissue disorders, NSSF and oral pH)	59 females diagnosed with ED *vs*. 120 females had no antecedents of ED, as healthy controls	Patients with EDs [DSM 5]	Oral hygiene habits recorded (frequency of dental check-ups, tooth brushing, mouthwash use, dental flossing, smoking habits and medication consumption). Dental variables analysed: degree of dental erosion; periodontal status (Ramfjord periodontal index-PI) and WHO periodontal probe; presence of caries (dental explorer probe and mirror); caries (the DMF- index and restoration index -RI); soft tissue lesions (exploration probes, mirror and gauzes). NSSF (draining technique). Salivary (pH Test Strips®)	Dental erosion was the most significant dental alteration observed, with its degree being notably higher in the ED group (*P* < 0·001). Significant association between soft tissue lesions and ED (*P* < 0·001). Marked significant difference in non-stimulated salivary flow between the groups (*P* < 0·001). No significant differences were found between the groups in terms of periodontal status, dental caries or oral hygiene practices.

ED, eating disorders; AN, anorexia nervosa; BN, bulimia nervosa; BED, binge eating disorder; EDNOS, eating disorder not otherwise specified; OSFED, other specified feeding and eating disorder; DSM, diagnostic and statistical manual of mental disorders; DMF, decay-missing-filled; EDI-2, Eating Disorder Inventory-2; VPI, visible plaque index; GBI, gingival bleeding index; DMFT, decayed, missing and filled teeth; DMFS, Decayed, missing, or filled surfaces; BEWE, Basic Erosive Wear Examination; UWS, unstimulated whole salivary; SWS, stimulated whole saliva; UKU, Udvalg for Kliniske Undersogelser; OHI-S, simplified oral hygiene index; GI, modified gingival index; BMD, bone mineral density; MCW, mandibular cortical width; TWI, tooth wear index; SIV, self-induced vomiting; VEDE, visual erosion dental examination; M.I.N.I., Mini International Neuropsychiatric Interview; PI, plaque index; BOP, bleeding on probing; CPI, community periodontal index; OHIP, oral health impact profile; OHRQoL, oral health-related quality of life; ALT, alanine aminotransferase, AST, aspartate aminotransferase; LDH, lactate dehydrogenase; TBARS, thiobarbituric acid reactive substance; PCR, plaque control record; GERD, gastroesophageal reflux disease; BMS, burning mouth syndrome; NSSF, non-stimulated salivary flow; DAI, dental aesthetic index; TMD, temporomandibular disorder; AMO, active mouth opening; PMO, passive mouth opening.

Finally, a dedicated section was included to examine the role and clinical approach of dental professionals in the context of ED (v. Dental Professionals’ Role and Approach). Particularly, emphasis was placed on the relevance of early-stage identification, highlighting how specific oral health assessments, such as enamel erosion, xerostomia and alterations in salivary flow and composition, may serve as early clinical indicators of ED onset. Furthermore, attention was given to the duration of illness when such data were available in the original studies, to explore potential correlations between disease progression and oral health outcomes.

### Caloric restriction and malnutrition

Caloric restriction is a hallmark behaviour in ED, particularly AN, where prolonged limitation of energy intake is driven by an intense fear of weight gain and a distorted body image^(51)^. This leads to deficiencies in macronutrients and micronutrients, disrupting metabolic and physiological functions and causing immune, hormonal and organ impairments^(51)^.

Malnutrition is worsened by increased physical activity, purging behaviours and gastrointestinal issues that reduce nutrient absorption, ultimately leading to muscle wasting, bone loss, cardiovascular problems and other severe health risks^(52)^.The caloric and nutritional deficits associated with AN lead to severe physical health complications, including growth retardation, osteopenia, amenorrhoea, renal insufficiency, abnormal laboratory values and dysfunctions in cardiac and thyroid systems^(14)^. The leading causes of death in individuals with AN are sudden cardiac death due to ventricular arrhythmias and suicide^(14)^; overall, mortality rates in AN are nearly six times higher than those in the general population^(53)^.

AN is strongly associated with a wide range of oral health complications, largely stemming from malnutrition caused by chronic caloric restriction and resulting nutritional deficiencies^(54)^, such as calcium, vitamin D and protein, which are critical for maintaining dental and periodontal health^(55)^. These nutritional deficits can contribute to weakened enamel, delayed dental development and increased susceptibility to periodontal disease^(56)^. For instance, as reported by Johansson *et al.*^(29)^, patients with ED, including AN, experienced significantly higher ORs for oral-related complications (OR = 4·1) such as burning tongue (OR = 14·2), dry or cracked lips (OR = 9·6), dental erosion (OR = 8·5) and reduced gingival bleeding (OR = 1·1), when compared with healthy controls. These findings were further confirmed by diverse studies included in this narrative review^(31–34,36–39)^. Notably, as most oral health clinical trials have focused on adult female patients, Paszynska *et al.*^(38)^ conducted a case–control study to investigate AN in younger individuals (<18 years old). They assessed dental health and gingival inflammation in a sample of 117 female adolescents diagnosed with the severe restrictive subtype of AN, comparing the results to those of a control group^(38)^. The study found that adolescents with AN exhibited poorer dental health than the controls, as indicated by a significantly higher decayed, missing and filled teeth score (*P* = 0·005); more severe erosive tooth wear (BEWE, *P* < 0·001); greater difficulty in plaque control (plaque control record, *P* < 0·001) and higher rates of gingival inflammation (bleeding on probing, *P* < 0·001); as well as a significant (*P* < 0·05) associations between gingival bleeding and erosive wear, plaque control record, decayed teeth and filled teeth with disease duration^(38)^. Identifying oral health issues associated with AN in adolescents is crucial due to the numerous dental complications that may arise with the restrictive subtype of the disorder since adolescence is a key period during which the onset of AN can lead to specific oral health issues, including an increased risk of dental caries, early stages of erosive tooth wear, gingival inflammation and difficulties in maintaining proper dental hygiene^(38)^. Early detection of these oral health problems can help prevent the progression of dental and periodontal conditions, which can otherwise contribute to further health complications and reduced quality of life in young patients with AN.

Reduced saliva production is another common effect of AN, arising from both malnutrition and dehydration, impairing the mouth’s inherent defence mechanisms against bacterial proliferation and heightening the vulnerability to dental caries and oral infections^(32)^. In the present review, three studies^(32,33,37)^ evaluated alterations in salivary biochemical parameters and flow rate, and oral health-related quality of life in patients with ED, including AN. Paszynska, *et al.*^(32)^. conducted a clinical case-control study on twenty-eight female adolescents with AN and thirty-eight age-matched controls, describing that salivary flow rates, both stimulated and unstimulated, were significantly lower in the AN group compared with controls (*P* = 0·0005 and *P* = 0·0001, respectively) and individuals with AN exhibited differences in the salivary enzyme activity of collagenase and aspartate aminotransferase activities in stimulated saliva (*P* < 0·05). The findings supported the hypothesis that reduced salivary flow may be a reliable indicator of AN, as patients exhibited decreased gland activity in both unstimulated and stimulated saliva^(32)^. Although key salivary enzymes maintain physiological activity despite malnutrition, the reduced salivary output likely lowers their overall concentration, indicating a partial physiological adaptation to severe malnutrition^(32)^. Similar findings were reported by Deghan *et al.*^(30)^, who, although not distinguishing among different ED, suggested that extensive tooth erosion in ED may be associated with low stimulated salivary flow, a result consistent with earlier studies by Lourenco *et al.*^(33)^ and Chiba *et al.*^(37)^.

### Purging behaviours: vomiting, laxatives and diuretics

Purging behaviours, including SIV, laxative and diuretic abuse, are common compensatory strategies observed across several ED. These behaviours are especially prevalent in BN and in AN, particularly the binge-eating/purging subtype, contributing to severe medical complications and nutritional imbalances^(56)^. The frequent exposure to stomach acid from vomiting erodes dental enamel, particularly on the inner surfaces of the teeth, leading to dental erosion; the acid exposure not only weakens enamel but also increases tooth sensitivity and the likelihood of dental fractures^(41)^. Over time, erosion can cause significant structural damage, contributing to functional impairment and aesthetic concerns^(57)^. In this context, Otsu *et al.*^(43)^ investigated the relationship between the severity of dental erosion and behaviours in individuals with ED, particularly focusing on daily diet and vomiting, in a sample of seventy-one patients (including those with AN). The study found that dental erosion was present in 86 % of patients who induced vomiting and significant differences were observed between those with mild and severe erosion, particularly regarding post-vomiting oral hygiene practices^(43)^. Patients with mild erosion were more likely to drink large amounts of water before vomiting, while those with severe erosion more frequently consumed carbonated beverages or sweetened foods^(43)^. The authors concluded that the severity of dental erosion in patients with ED is influenced by vomiting-related behaviours and the habitual consumption of specific foods and beverages^(43)^.

Again, SIV or excessive AN, combined with reduced salivary flow, poor oral hygiene and nutritional deficiencies, further weakens the oral mucosal defences, making the mouth more susceptible to infections^(25)^; vomiting exposes the oral cavity to gastric acid, which can erode tooth enamel, leading to dental erosion and increased vulnerability to bacterial colonisation^(31)^. Esteves *et al.*^(45)^ conducted a cross-sectional, case-control study to investigate the prevalence of oral Candida species in individuals with ED, including AN. The authors concluded that alterations in the natural oral and gut microbiota, particularly due to purgative behaviours, may predispose patients to the acquisition of non-albicans species, such as *C. glabrata* (commonly found alongside *C. albicans* in oropharyngeal candidiasis), *C. parapsilosis* (often isolated from the subungual space of human hands) and *C. dubliniensis* (a normal component of the upper respiratory tract flora). Unlike *C. albicans*, these species may contribute to the development of oral candidiasis in individuals with ED^(45)^.

BN is strongly associated with oral health issues, primarily due to the recurrent SIV and dietary habits associated with the disorder^(41)^.

Over time, acidic exposure weakens the enamel, leading to dental erosion, increased tooth sensitivity and a higher risk of cavities^(33,34)^. Among the studies included in this narrative review, Manevsky *et al.*^(48)^ investigated the presence, localisation and severity of dental erosion using the Basic Erosive Wear Examination (BEWE) index and assessed the decayed, missing and filled teeth index in thirty patients purging BN and thirty healthy controls. The study employed both questionnaires and clinical examinations to collect data^(48)^; the results revealed that dental erosion was significantly more prevalent and severe in patients with purging BN compared with controls (*P* < 0·05), particularly on the oral surfaces of the teeth which were most affected, showing significant wear and thinning of the enamel^(48)^. However, at the same time, the decayed, missing and filled teeth index values did not differ significantly between the two groups (*P* = 0·461)^(48)^.

In individuals with BN, salivary glands may also swell due to chronic vomiting, leading to a visible enlargement of the glands near the jaw, giving the face a swollen appearance^(58)^. Besides, repeated acid exposure can alter the composition of saliva, reducing its protective properties and promoting a dry mouth environment, which can increase the likelihood of dental decay, halitosis and infections^(59)^. Among the studies examined, Dynesen *et al.*^(35)^ analysed the impact of BN on the functionality of the salivary glands and the correlation with dental erosion. This case–control study, evaluating twenty female subjects diagnosed with BN according to the DSM-IV and twenty healthy subjects, reported that unstimulated whole saliva flow was significantly lower in the BN group compared with controls (*P* = 0·007), with the frequency of SIV and binge eating having no impact on the unstimulated whole saliva flow rate^(35)^. The duration of the ED was inversely related to the salivary flow rate (*P* = 0·019) in the BN group, and hyposalivation (unstimulated whole saliva ≤ 0·1 ml/min) was more common in the BN group, as patients reported a significantly greater sensation of dry mouth (*P* = 0·003)^(35)^. Additionally, oral dryness and dental erosion were significantly more pronounced in the BN group (*P* = 0·003 and *P* = 0·019, respectively), with a significant correlation between the duration of the ED and the severity of dental erosion (*P* = 0·007)^(35)^. Similar conclusions were also reported by studies included in the present review^(42,44,48)^.

At the same time, an interesting cross-sectional study conducted by Blazer *et al.*^(44)^ examined the salivary composition and taste perception in twenty-six female patients with BN, and 26 were well-matched for age and BMI controls, starting from the a priori hypothesis that changes in either taste perception or salivary composition might be associated with the active disease. In this context, examinations showed an overall disturbed salivary and taste profile in patients, who complained of xerostomia (dry mouth, *P* < 0·003) and taste aberration or oral burning sensation (*P* < 0·016)^(44)^. Based on the findings of this study, the authors speculated that disturbances in salivary and taste profiles in patients with BN, regardless of the underlying mechanism, could serve as valuable diagnostic tools and enhance understanding of the disease’s pathogenesis, as these disruptions may potentially trigger or exacerbate the ED^(44)^. However, due to the lack of a significant correlation between these disturbances and clinical manifestations, as well as the small and heterogeneous patient sample, further research with larger cohorts is necessary to determine whether these disturbances normalise with recovery^(44)^. If normalisation occurs, therapeutic interventions such as antioxidants, anti-inflammatory drugs and saliva substitutes – administered both locally and systemically – should be considered for individuals with BN^(44)^.

Again, regarding the salivary composition, Chiba *et al.*^(37)^, in a quantitative cross-sectional study, investigated periodontal health, alterations in salivary biochemical parameters and oral health-related quality of life in patients with ED, including BN (thirty women with ED *v*. 30 women as healthy controls). Data showed that the ED group had markedly higher salivary concentrations of total protein (*P* = 0·0109), aspartate aminotransferase (*P* = 0·0108), alanine aminotransferase (*P* = 0·0455) and lactate dehydrogenase (*P* = 0·0252) than controls, while no differences were found in salivary thiobarbituric acid reactive substance levels^(37)^. Furthermore, Aframian *et al.*^(46)^ in a cross-sectional and case–control study, compared the pH of oral mucosa of healthy subjects to individuals with gastroesophageal reflux disease, BN and burning mouth syndrome^(46)^. In this case, both the BN and gastroesophageal reflux disease groups demonstrated significantly lower pH levels (6·38 ± 0·45 and 6·51 ± 0·32, respectively) compared with the control group (6·8 ± 0·33)^(46)^. The present narrative review included six studies involving subjects with EDNOS^(29,31,34,41,43,47)^; among them, the only study in which the oral health characteristics of subjects with EDNOS can be described is the study by Panico *et al.*^(41)^, as the other studies do not discriminate between EDNOS and other ED. Therefore, this prospective case-control study aimed at describing oral lesions in women with ED (*n* 65), also including subjects with EDNOS (*n* 13), together with subjects with AN (*n* 6), as well as BN (*n* 46). In particular, the presence of all oral lesions combined was associated with purging behaviours (OR = 6, 95 % CI = 1·06, 34·12, *P* = 0·0414) and labial erythema was more frequent in individuals with BN than in those with AN or EDNOS (*P* = 0·0098) and linked to SIV (OR 4, 95 % CI 1·08, 14·77, *P* = 0·0396) and diuretic/laxative use (OR 7·89, 95 % CI 2·18, 28·56, *P* = 0·0012)^(41)^. However, the oral examination revealed that 43 % of the patients with EDNOS displayed exfoliative cheilitis and an orange-yellow palate^(41)^; additionally, haemorrhagic lesions and instances of cheek or lip biting were observed^(41)^.

### Binge eating episode

Binge eating episodes, characterised by the compulsive consumption of large amounts of food within a discrete period accompanied by a sense of loss of control, are commonly described in both BN and BED^(1)^. In BN, these episodes are typically followed by compensatory behaviours such as SIV, laxative or diuretic abuse, whereas in BED, compensatory behaviours are absent^(1)^. Both disorders share the common behaviour of excessive intake of carbohydrate-rich, sugary and acidic foods during binge episodes, which have significant implications for oral health. Although data specifically focusing on BED in the context of oral health remain limited – Uhlen *et al.*^(31)^ included only one patient with BED in their cohort – the interplay between binge eating episodes and oral pathology is relevant across both BN and BED populations. The frequent consumption of cariogenic and acidic foods leads to repeated acid attacks on dental enamel, increasing the risk of dental erosion and caries development^(29)^. The acids naturally present in foods consumed during binge episodes (such as fruit juices and citrus fruits) can contribute to enamel demineralisation and erosion^(60)^. While dental erosion affecting the palatal surfaces is more commonly associated with vomiting, buccal and facial surfaces may also be affected in patients who consume excessive amounts of acidic foods, particularly raw fruits with high acid content^(60)^. This repeated exposure to sugars and acids disrupts the oral environment, fostering demineralisation and weakening of enamel, thus increasing tooth sensitivity and vulnerability to structural damage. Johansson *et al.*^(34)^ investigated binge eating and SIV behaviours in individuals with ED, reporting that in fourteen out of seventeen cases, binge eating preceded vomiting episodes. The binge episodes typically involve high-calorie, sugar- or fat-rich and acidic foods and drinks, which exacerbate oral acid exposure and enhance enamel erosion and caries risk^(34)^. Among individuals with ED, those with BED appear to be particularly susceptible to an increased risk of dental caries. This may be due to the frequent intake of high-calorie, carbohydrate-dense foods, often consumed in large quantities and at high frequency throughout the day. A higher frequency of food intake throughout the day may lead to an augmented risk of developing dental caries^(60)^. Moreover, repeated binge eating and compensatory vomiting can result in soft tissue injuries, including trauma to the oral mucosa and pharyngeal tissues. These injuries may stem from the mechanical stress of rapid food intake^(60)^. Within-group analyses showed that ED patients with present vomiting and/or binge eating behaviours perceived their oral health as significantly worse (OR = 6·0) and exhibited a higher risk of dental erosion (OR = 5·5) compared with those without such behaviours^(29)^.

### Oral hygiene neglect

Poor oral hygiene may further exacerbate the oral health complications associated with ED, acting as an additional and often overlooked risk factor^(61)^. Individuals with ED, particularly those with AN or BN, frequently experience a combination of psychological distress, social withdrawal and physical fatigue that can lead to the neglect of routine oral hygiene practices and avoidance of regular dental check-ups^(61)^. This neglect, compounded by the systemic effects of malnutrition or repeated exposure to gastric acid in purging behaviours, creates a synergistic effect that accelerates the deterioration of oral tissues^(61)^. For example, inadequate plaque control and prolonged exposure to acidic environments can hasten enamel erosion, exacerbate gingival inflammation and increase the risk of caries and periodontal disease^(38,49)^. Moreover, the stigma surrounding ED may prevent individuals from disclosing their condition to dental professionals, further delaying appropriate preventive or therapeutic interventions^(61)^. Therefore, poor oral hygiene should be considered not only because of ED but also as a modifiable co-factor contributing to the progression of ED-related oral pathology.

However, there is an apparent discrepancy between clinical assumptions mentioned and patient-reported behaviours, highlighting the complexity of the relationship between ED and oral hygiene. The existing literature suggests that women with ED often show enhanced oral hygiene practices and attitudes towards dental visits comparable to the general population^(36)^. A survey conducted in 2016 involving thirty patients with ED found that 40 % of patients brushed their teeth more than twice daily, compared with 17·24 % of controls (*P* = 0·01)^(50)^. Regarding oral hygiene after the onset of illness, 56·67 % of patients reported an increase in oral care, while 40 % reported no change in their routine^(50)^. Additionally, when inquired about their oral hygiene habits following the onset of their illness, 56·67 % of patients indicated that they engaged in more diligent oral care^(50)^.

Several factors may help reconcile this discrepancy. On one hand, self-reported improvements in oral hygiene may reflect compensatory behaviours, particularly among individuals with BN who may attempt to mitigate the perceived effects of vomiting on dental health. On the other hand, the psychological profile of certain individuals with ED, marked by perfectionism and obsessive-compulsive traits^(60)^, may drive more frequent hygiene routines, potentially masking deeper underlying oral pathology. Finally, enhanced frequency of toothbrushing, especially immediately after purging episodes, may paradoxically contribute to dental erosion due to mechanical abrasion of already acid-weakened enamel.

### Dental professionals’ role and approach

To effectively address the clinical conditions of the oral cavity in patients with ED, a proactive, comprehensive and personalised approach involving dental practitioners and dental hygienists is indispensable. This strategy emphasises the importance of preventive care, early detection and multidisciplinary collaboration, focusing on the unique oral health challenges faced by this patient population. Defining ‘early-stage’ ED is crucial to optimise timely intervention and improve clinical outcomes. Recent staging models characterise early stages by the emergence of initial symptoms that may not yet fulfil full diagnostic criteria for severe or enduring illness^(62,63)^. These stages can be identified through targeted oral health assessments revealing early clinical signs such as enamel erosion, mild gingivitis, xerostomia and nutritional deficiencies. Recognition of these oral manifestations enables dental practitioners to detect ED promptly, facilitating intervention before disease progression and supporting integration within a multidisciplinary treatment framework^(62,63)^.

Dental practitioners should prioritise regular monitoring and individualised care plans for patients with ED^(49)^. Scheduling periodic recall visits is essential to reducing the incidence of periodontal and dental lesions^(49)^. A check-up serves as a cornerstone for this approach, enabling the systematic assessment of critical parameters, including the plaque index, gingival recession, and the BEWE index^(49)^. These indicators facilitate the early detection of pathological changes and allow for timely interventions.

The prevention of secondary lesions requires the effective removal of bacterial plaque retention factors, which is particularly significant for patients with systemic conditions associated with ED^(30)^. Salivary dysfunction, a common complication in these patients, diminishes the natural buffering capacity of saliva, increasing oral acidity and heightening the risk of carious lesions. This vulnerability is reflected in elevated scores on the decayed, missing and filled teeth index^(30)^. Dental professionals must address these risk factors through meticulous plaque control and tailored therapeutic interventions^(30)^.

Initial evaluations should focus on determining each patient’s susceptibility to dental erosion, emphasising the assessment of local risk factors such as plaque accumulation^(64)^. Preventive measures, including remineralisation therapy and dietary counselling, should be integrated into a broader therapeutic framework. This requires collaboration with dieticians and nutritionists to develop a nutritional therapeutic plan aimed at mitigating the progression of dental erosion. Remineralisation protocols should be individualised based on the patient’s degree of susceptibility, as determined by the BEWE index.

The BEWE index provides a standardised methodology for quantifying dental erosion, categorising enamel loss on a scale from 0 (no enamel loss) to 3 (loss exceeding 50 % of the tooth surface)^(64)^; the highest score within each sextant of the dentition is recorded, and the total BEWE score is calculated by summing these values^(64)^. Based on the cumulative score, patients are classified into four categories of susceptibility: no susceptibility (0–2), low susceptibility (3–8), medium susceptibility (9–13) and high susceptibility (≥14)^(64)^. This classification informs the duration and intensity of remineralisation protocols, which range from two months for low susceptibility to 6 months for high susceptibility^(64)^. Biomimetic hydroxyapatite-based substances are recommended for these treatments due to their efficacy in enamel restoration and resistance to further erosion^(64)^.

Dental erosion compromises the enamel layer, exposing the underlying dentin and its associated nerve endings, thereby increasing dentin hypersensitivity^(65)^. To evaluate the success of remineralisation therapy, clinicians should employ the Schiff air index, a diagnostic tool that quantifies patient sensitivity to air stimulation. Scores range from 0 (no response) to 3 (severe pain and withdrawal), providing an objective measure of therapeutic outcomes and patient progress^(65)^.

Recent advances in teledentistry have expanded the potential for remote patient monitoring and intervention, where using specialised applications, patients can submit self-assessments and images for professional evaluation^(66)^. This technology supports the ongoing management of dental, periodontal and nutritional conditions, offering a practical solution for maintaining care continuity between in-person visits^(66)^. Such approaches allow dental practitioners to extend their involvement beyond traditional clinical settings, ensuring comprehensive and accessible care for individuals with ED^(66)^. Therefore, the integration of these methods underscores the pivotal role of dental professionals in the multidisciplinary management of ED, highlighting the necessity of continuous education, innovative technologies and collaboration to optimise patient outcomes.

## Discussion

The findings presented in this narrative review highlight the complex and multifactorial relationship between ED (particularly, AN, BN, BED and OSFED) and oral diseases, with significant implications for patients’ nutritional status and overall health.

Chronic caloric restriction and associated malnutrition, hallmarks of AN and BN, play a central role in the pathophysiology of oral complications^(67)^, with widespread micronutrient deficiencies (e.g. Ca, vitamin D, vitamin C and phosphate)^(55,56)^, as well as a lack of protein and healthy fats^(55,68)^ which can disrupt tooth mineralisation^(69)^ and weaken the bones supporting the teeth, increasing the risk of decay and impairing the homeastasis, such as structural integrity and resilience of the oral cavity^(56,68)^. Again, for patients with AN, severe caloric restriction is associated with dry mouth (xerostomia) and gingivitis (primarily due to vitamin C and calcium deficiencies) that weakens the gums and increases infection risk^(29,30,32,33,38)^. Finally, individuals with AN or BN may experience edentulism (tooth loss), an outcome that, while uncommon, underscores the profound systemic effects these disorders can have, including their impact on oral health^(70)^. The development of edentulism in this context is primarily driven by behaviours and physiological changes associated with disorders that directly or indirectly harm the integrity of teeth and supporting structures^(71)^.

This imbalance often perpetuates disordered eating behaviours, as painful or difficult chewing makes mealtime distressing, leading to avoidance of necessary foods^(68)^. The cumulative effects of these oral health complications lead to discomfort, pain and functional limitations, making eating an unpleasant experience and reinforcing disordered eating behaviours^(72)^.

The systemic effects of malnutrition are compounded by purging behaviours (e.g. SIV and laxative or diuretic abuse) related to body image and self-care habits^(41)^, thus introducing additional oral stressors, such as acid erosion, salivary dysfunction and increased susceptibility to oral infections^(25,30,32)^. One of the most significant contributors is gastric acidity, particularly in individuals with BN who engage in SIV^(31)^. Repeated contact with gastric contents leads to progressive enamel erosion, weakening the tooth structure, increasing fragility, promoting gingival inflammation, reducing salivary flow and raising the risk of tooth loss^(35,42,44,48)^. These effects are exacerbated by the frequent consumption of sugary and acidic foods, especially among individuals experiencing binge eating episodes, which significantly increase the risk of dental caries^(43)^. In fact, in BED, the habitual intake of simple carbohydrates and fermentable sugars fosters the proliferation of cariogenic bacteria, thereby heightening the likelihood of both tooth decay and periodontal disease^(34)^. Similarly, patients diagnosed with OSFED may also exhibit oral manifestations such as dental erosion and gingivitis, particularly in the presence of binge eating or purging behaviours^(41)^. Reduced salivary flow exacerbates vulnerability to dental caries and periodontal diseases, further increasing the likelihood of tooth loss^(73)^. Similarly, ED increase the risk of gingival and periodontal diseases, which, exacerbated by poor oral hygiene, nutritional deficiencies and systemic inflammation, can lead to tissue destruction and tooth loss if untreated^(74)^.

An intriguing and paradoxical finding arises from the analysis of oral hygiene behaviours. While clinical assumptions have long suggested a general neglect of oral hygiene in individuals with ED^(25,61)^, some self-reported data^(36,51,60)^ contradict this view, particularly among individuals with BN. Enhanced oral hygiene practices may reflect compensatory routines aimed at mitigating the effects of vomiting, or may be influenced by personality traits such as perfectionism or obsessive-compulsiveness. However, these behaviours (especially toothbrushing immediately after purging) may inadvertently accelerate enamel erosion due to mechanical abrasion of acid-weakened surfaces.

Therefore, as oral health declines, the capacity to maintain a balanced diet diminishes, which exacerbates both nutritional imbalances and the physical manifestations of ED^(70)^. Further, deteriorating oral health can affect social interactions and self-esteem, intensifying the psychological burden of ED^(57)^.

The present review also highlights the critical role of dental practitioners and hygienists in the early identification and multidisciplinary management of ED. Dental professionals are often among the first to observe early manifestations of ED, such as enamel erosion, gingival inflammation or xerostomia^(27)^, and they are uniquely positioned to initiate timely referrals and preventive interventions or supporting recovery efforts^(27)^. The use of clinical indices such as BEWE^(75)^ and Schiff air index^(76)^, alongside teledentistry tools^(77)^, can enhance early detection and longitudinal monitoring of oral health status, particularly in vulnerable or hard-to-reach populations.

By addressing oral diseases within the context of ED management, healthcare professionals can alleviate certain physical barriers to nutrition, supporting a comprehensive recovery that encompasses the physiological, nutritional and psychological complexities of ED.

Reduced salivary flow exacerbates vulnerability to dental caries and periodontal diseases, further increasing the likelihood of tooth loss^(73)^. Similarly, ED increase the risk of gingival and periodontal diseases, which, exacerbated by poor oral hygiene, nutritional deficiencies and systemic inflammation, can lead to tissue destruction and tooth loss if untreated^(74)^.

Overall, the findings described in the present narrative review highlight the importance of a multidisciplinary approach that includes oral health in the treatment of ED, aiming to enhance early diagnosis and management of oral complications. However, it is interesting to note that although a growing body of literature examines the impact of ED on oral health, significant gaps remain, particularly regarding diverse demographic representation. Most studies reviewed focus on women and younger individuals, overlooking potential variations in ED-related oral health effects among men and older adults^(30,32,33,35–37,39–45,47,49,50)^. Additionally, inconsistent diagnostic criteria^(29,31,35,37,39,48,50)^ contribute to discrepancies in findings, hindering definitive conclusions. Finally, despite advancements, the high prevalence of ED comorbid with other psychological conditions remains underexplored, potentially leading to the neglect of comprehensive care strategies^(78)^. While meta-analyses^(79)^ confirm the efficacy of treatments for ED, a substantial group of patients shows inadequate response to existing therapies^(80)^.

Therefore, future research should address these limitations, focusing on understanding and addressing the needs of this overlooked group, thereby improving prevention and treatment strategies tailored to individual needs.

### Conclusions

The interplay between ED and oral health is driven by a combination of nutritional, behavioural and psychosocial factors. An integrated care model, encompassing dental, medical, nutritional and psychological expertise, is essential for mitigating oral health risks and improving overall outcomes for individuals with ED.

This review emphasises the critical need for routine dental screenings in at-risk populations, where early detection of oral manifestations, such as enamel erosion, xerostomia and increased caries, may reveal underlying disordered eating behaviours.

Advocating for a collaborative, interdisciplinary model, this review underscores the necessity of integrating dental and psychological care into a more holistic treatment approach. Addressing ED in this manner not only targets primary psychological drivers but also addresses the systemic and often severe physiological repercussions of these disorders, especially in the oral cavity, where effects can be particularly profound. By coordinating nutritional management with dental treatment, healthcare providers can create a cohesive care plan that enhances the efficacy of both psychological and physiological interventions. This integrative model strives to deliver timely and tailored care that enhances patients’ quality of life through early diagnosis and prompt intervention. Additionally, it emphasises the importance of increasing awareness among healthcare professionals, who must recognise the link between oral health deterioration and ED. By fostering a deeper understanding of this connection, healthcare providers are better equipped to identify, support and manage at-risk individuals, contributing to improved patient outcomes and a more comprehensive healthcare framework.
